# Antibodies, synthetic peptides and related constructs for planetary health based on green chemistry in the Anthropocene

**DOI:** 10.4155/fsoa-2017-0101

**Published:** 2018-01-10

**Authors:** Salvador Eugenio Caoili

**Affiliations:** 1 Department of Biochemistry & Molecular Biology, College of Medicine, University of the Philippines Manila, Room 101, Medical Annex Building (Salcedo Hall), 547 Pedro Gil Street, Ermita, Manila 1000, Philippines

**Keywords:** antibodies, chemically programmable immunity, drugs, epitopes, green chemistry, peptide-based vaccines, planetary health, precision health, synthetic peptides, xenobiotics

## Abstract

The contemporary Anthropocene is characterized by rapidly evolving complex global challenges to planetary health vis-a-vis sustainable development, yet innovation is constrained under the prevailing precautionary regime that regulates technological change. Small-molecule xenobiotic drugs are amenable to efficient large-scale industrial synthesis; but their pharmacokinetics, pharmacodynamics, interactions and ultimate ecological impact are difficult to predict, raising concerns over initial testing and environmental contamination. Antibodies and similar agents can serve as antidotes and drug buffers or vehicles to address patient safety and decrease dosing requirements. More generally, peptidic agents including synthetic peptide-based constructs exemplified by vaccines can be used together with or instead of nonpeptidic xenobiotics, thus enabling advances in planetary health based on principles of green chemistry from manufacturing through final disposition.

## Lay abstract

Radical change in the role of humans as planetary custodians is necessary for the long-term well-being of all life. Drugs currently in use and under development tend to be foreign substances whose effects on both body and environment are difficult to predict. Antibodies and related molecules can lessen the safety hazards posed by said drugs, in part by decreasing the requirement for drug intake. More generally, proteins and peptides can be produced and used (notably as vaccine components) in line with green (i.e., eco-friendly) chemistry to better address health needs, alongside or even in place of said drugs.

The contemporary Anthropocene (i.e., geological epoch of significant human impact on the biosphere) entails rapidly evolving global health challenges due to radical socio-environmental transformations driven by ever-accelerating technological change [1]. These confound initiatives toward planetary health (i.e., well-being of the entire biosphere) and sustainable development (i.e., meeting present needs without depriving future generations of means to meet theirs). Adaptive innovation is thus urgently required for long-term survival and flourishing of human civilization, yet it is subject to the prevailing precautionary regime of regulations that has arisen in response to undesirable outcomes (e.g., environmental contamination) associated with technological change (e.g., widespread use of xenobiotic chemical entities in agriculture, industry and healthcare). This tension between imperative to innovate and attendant risk aversion has been addressed in a previously outlined framework as regards drug development [2], albeit mainly in a limited sense of human safety at the individual level, without sufficient attention to wider ecological consequences. Hence, this commentary aims to extend the earlier framework and thereby provide a more holistic view of options for advancing health through the use of antibodies, synthetic peptides and related peptidic agents.

## Safety & sustainability

Safety embodies the bioethical principle of nonmaleficence (i.e., minimizing harm in pursuit of benefit). In traditional drug development, individuals are exposed to a candidate or clinically approved drug and hence are at risk for adverse outcomes (e.g., death or disability). A more proactive approach thus would be deliberate development of drugs and antidotes thereto in tandem throughout the entire translational pipeline (i.e., from basic research through clinical trials to regulatory approval for routine use), with antibody-type agents (i.e., antibodies and derivatives thereof, such as Fab and scFv fragments) serving as antidotes (e.g., via noncovalent sequestration or even catalytic degradation) and related drug-action modulators (e.g., drug-concentration buffers that enable lower and less frequent dosing) [2]. Still, this framework *per se* fails to comprehensively address safety vis-a-vis sustainability, herein defined as resource utilization compatible with supporting planetary health indefinitely.

In the long run, safety depends on sustainability. Accordingly, international efforts aim to curb anthropogenic greenhouse gas emission and thereby decrease the overall human carbon footprint (i.e., amount of emitted carbon due to fossil fuel consumption) [3]. This quantity comprises contributions due to processes associated with the life cycle (comprising creation, storage, distribution, use and disposal) of various products (e.g., food, drugs and devices); and a carbon footprint thus may be estimated per product as an indicator of sustainability, with each product ideally being carbon neutral (i.e., having zero carbon footprint, as might be achieved for biofuels produced via photosynthetic carbon fixation). More generally, life cycle assessment (LCA) may be performed on each product to account for all consumed resources (i.e., energy and materials) and generated byproducts (e.g., waste heat and chemical pollutants) throughout the product life cycle [4]. LCA thus enables much more informative product evaluation than simple estimation of a single carbon footprint value, in that different carbon footprint values can be obtained for alternative scenarios (e.g., using fossil fuel versus renewable energy sources) while byproducts other than atmospheric carbon are explicitly considered in relation to environmental impact (e.g., direct toxicity of chemical pollutants, apart from greenhouse gas activity).

## From thermodynamics to nonmaleficence via green chemistry

LCA frames the development of green (i.e., sustainable) chemistry, which comprises the theory and practice of both chemistry and chemical engineering to support sustainability. In essence, resource utilization can be equated with energy utilization insofar as resources are forms of energy, which subsumes all matter according to the mass–energy equivalence relationship. All this is constrained by thermodynamics, of which the first law asserts the conservation of energy (whereby total energy remains constant over time) while the second (asserting that total entropy tends to increase over time) implies that the efficiency of energy-transformation processes (e.g., chemical reactions) tends to be imperfect (i.e., with at least some energy rendered unavailable for performing useful work). These laws underlie the principles of green chemistry, which can be largely rationalized in terms of conservation and efficiency, particularly with reference to atom economy (i.e., maximizing the incorporation of atoms from chemical reactants into desired products rather than unwanted byproducts) and step economy (i.e., maximizing the efficiency of chemical processes by minimizing the number of steps per process, noting that losses are inevitably incurred with each step) [5]. At a systems level, green chemistry is envisioned to support a circular economy wherein product life cycles form closed loops of efficient resource utilization analogous to naturally occurring biogeochemical (e.g., carbon, nitrogen and phosphorous) cycles, with LCA viewed as cradle-to-cradle rather than cradle-to-grave analysis (i.e., with all processes yielding products that are in turn inputs for other processes, such that all products are renewable resources).

Beyond chemical processes *per se*, green chemistry is broadly concerned with minimizing harm due to chemical entities. Such harm is epitomized by global warming due to atmospheric greenhouse gases, for which reason efficient resource utilization in general is key to sustainability; but more direct threats (e.g., toxic and explosion hazards) also must be addressed. Green chemistry thus aims to minimize harm due to various reactants, intermediates, desired products, byproducts, solvents, catalysts and other materials that present occupational, environmental and other safety hazards during various phases of product life cycles, noting that all said materials eventually undergo chemical transformation (e.g., upon degradation). As regards the pharmaceutical industry in particular, safety concerns thus arise in relation to manufacturing and downstream processes through the consumption of pharmaceutical products and consequent environmental contamination; yet traditional drug development is more narrowly focused on effects of drugs and their metabolites within the body, neglecting the impact of substances released into the environment [6].

Pharmaceutical manufacturing involves traditional synthetic chemical approaches, which heavily utilize hazardous substances (e.g., organic solvents), and also biotechnological approaches with quality-control issues (e.g., microbial contamination) arising from the complexity of biological systems (e.g., which can exhibit chaotic behavior insofar as they are exquisitely sensitive to environmental perturbations). Apart from industrial accidents and similar mishaps, deliberate misuse of manufacturing capacity (e.g., by rogue states and terrorist organizations, for chemical or biological attacks) further complicates the discourse on safety and related issues of security, notably with regard to dual-use technology with both peaceful and military potential (e.g., supporting beneficial economic activities versus stockpiling weapons of mass destruction). Such threats are addressed by civil defense (i.e., protection of civilian populations from military action and natural disasters) as articulated in the concept of CBRNE (chemical, biological, radiological, nuclear and explosives) defense [7], which could be facilitated by replacement of many currently employed technologies by less hazardous alternatives based on green chemistry.

Further downstream, drugs and metabolites thereof exert biological effects that impact both patient and environment. Mass consumption of drugs (e.g., as anti-infectives and maintenance therapy for chronic conditions) is enabled by facile chemical synthesis, stability at ambient temperatures and high oral bioavailability, all of which are typically realized simultaneously with small-molecule xenobiotic drugs; yet such drugs pose difficulties for prediction of pharmacodynamics, pharmacokinetics and, consequently, patient safety, let alone environmental impact of the drugs and their metabolites following excretion. Moreover, effects on the environment generate feedback that compromises safety. As a case in point, excreted antimicrobials impose selection pressure that drives emergence and spread of drug resistance among microbial populations in the environment, leading to drug-resistant infections in a vicious circle reinforced by current prescribing practices in both human and veterinary medicine, perhaps best exemplified by the agricultural use of antibiotics on a massive scale to hasten livestock growth and increase the yield of animal biomass per unit feed input at the expense of the gut microbiota [8]. On a related note, antibiotic-mediated suppression of the normal microbiota especially during early developmental stages (e.g., infancy) contributes to immune dysregulation that underlies increased incidence of allergic and autoimmune disorders as societies undergo modernization [9]. In theory, potentially hazardous excreted drugs and drug metabolites could be sequestered and rendered inactive (much as medical radioisotopes are recovered from patients and stored as radioactive waste with proper containment until the radioactivity has decayed to some acceptable level); but this would entail significant additional expenditure of resources and thus still detract from sustainability.

## Peptidic agents for planetary & precision health

Current and anticipated planetary-health challenges conceivably can be met by shifting the emphasis of drug development toward peptidic agents (i.e., peptides and proteins) that biodegrade into the 20 canonical proteinogenic amino acids, which are naturally occurring metabolites among all known forms of life. Such peptidic agents could complement and even largely supplant nonpeptidic small-molecule xenobiotics that dominate the current repertoire of approved and candidate drugs, in a manner consistent with principles of green chemistry as regards manufacturing and consumption of pharmaceutical products. This would be achieved primarily by adopting an immunocentric (i.e., immunity-based) approach to disease control and prevention, reminiscent of early modern biomedical research in its emphasis on vaccines and immunization yet fully exploiting contemporary scientific and technological advances from an ecologically motivated holistic systems perspective, in pursuit of planetary health sharpened by precision health (i.e., customized health intervention for individuals that is appropriate for their unique circumstances including genomic characteristics, developmental history and environmental exposure).

Enhancement of immunity (i.e., resistance to disease, mediated by the host immune system) is key to avoiding dependence on xenobiotic drugs. This is obvious with the classic use of vaccines for prophylactic active immunization against infectious diseases, which obviates anti-infective drug therapy and thereby avoids associated problems of emerging drug-resistant infections and disrupted microbiota. More generally, vaccines can be developed and used for both prophylaxis and treatment of infectious and noninfectious (e.g., neoplastic, inflammatory and endocrine) conditions [10]. Historically, vaccines were first developed as complex biologics (e.g., killed or live-attenuated pathogens, contaminated by host- or culture-derived impurities), of which certain components presented significant safety issues (e.g., injurious inflammatory responses to highly immunogenic vaccine antigens, reversion of attenuated strains to virulent forms, and allergic reactions to impurities); but these issues can be circumvented by careful vaccine formulation with selective inclusion of vaccine antigens by recombinant or even synthetic chemical means, as is feasible for peptide-based vaccines comprising only selected epitopes (i.e., submolecular structural determinants recognized by individual immune-system components) rather than whole-protein antigens containing deleterious epitopes (e.g., that can elicit autoimmune responses) [11]. Active immunization with peptide-based vaccines thus can elicit immune responses that target only particular epitopes, via selective activation of epitope-specific T cells (which can directly kill abnormal cells or coordinate wider cell-mediated immune responses involving other cell types such as macrophages) and production of antibodies (which can directly disrupt target function or activate effector mechanisms such as complement pathways for target elimination). The capability to selectively target only particular epitopes supports precision health (e.g., for immune-mediated cancer therapy, considering that useful target epitopes vary widely among patients due to both cancer mutations and host genetic polymorphisms).

Immunity mediated by antibodies and other antibody-type agents can be directed to a wide variety of chemically diverse (i.e., peptidic and nonpeptidic) targets and rapidly established via passive immunization (e.g., by administration of exogenous antibodies). As already noted in the context of patient safety, antibody-type agents can serve as antidotes and other drug action modulators, in particular as drug-concentration buffers that enable lower and more frequent dosing, which in itself supports sustainability in the sense of economizing on resources; yet, it also limits drug and drug metabolite excretion, thereby decreasing environmental contamination. Furthermore, synthetic peptide-based constructs may be designed as immunological adaptors that are bound by preformed antibody-type agents and physically link them to particular targets (e.g., pathogens) for rapid programming of immune responses, again supporting sustainability by repurposing the said agents instead of generating novel alternatives (albeit at the comparatively lower cost of generating novel adaptors) [12]. This also could support precision health by conferring immunity to specific targets (e.g., emerging pathogens for which anti-infective drug prophylaxis or therapy still might be unavailable) essentially on demand (e.g., during an epidemic).

Peptide-based constructs and antibody-type agents thus form a basis for better aligning the pharmaceutical industry and the wider health sector with the overall goal of sustainability. Peptide-based constructs can serve as vaccines, immunological adaptors and even full-fledged drugs, with their chemical synthesis obviating dual-use and other problems associated with biotechnology. Moreover, shifting the focus of chemical synthesis from nonpeptidic to peptidic products provides opportunities to simplify the bulk of pharmaceutical manufacturing processes in line with green chemistry, which is increasingly applied to peptide synthesis (e.g., with replacement of organic solvents by aqueous solutions) [13]. At the same time, antibody-type agents can be derived from human volunteers (e.g., healthy vaccinees) and possibly also produced via refinement of peptide-synthesis methodology to enable synthesis and proper folding of sufficiently long chains, again circumventing problems of biotechnology. Both peptide-based constructs and antibody-type agents can be prepared in forms (e.g., lyophilized material) suitable for storage at ambient temperatures rather than requiring cold-chain infrastructure, which is indispensable for traditional biologics and a major barrier to sustainability [14]. Inefficient delivery of both peptide-based constructs and antibody-type agents *in vivo* still hinders their wider application, as peptidic agents tend to be degraded via digestive processes in the gut, with difficulty of delivery increasing with molecular size; yet these problems can be addressed by less invasive parenteral delivery modes (e.g., using microneedle-array patches, as an alternative to syringes and hypodermic needles [15]) and even oral delivery for transport across the gut mucosa (e.g., using liposome-based and other carrier systems [16]). As peptidic agents are subject to enzyme-catalyzed hydrolytic degradation in the body (e.g., in the blood plasma, liver and kidneys) and the external environment (e.g., due to microbial populations), they are metabolized into progressively smaller fragments, yielding amino acids that are biologically assimilated in nature without complications (e.g., environmental persistence and neuroendocrine disruption) that characterize xenobiotics.

## Translational development of peptidic agents

Peptide-based vaccine development is supported by immunoinformatics (i.e., bioinformatics applied to immunology), which provides means for rapid large-scale analyses of peptidic sequence and structure data (e.g., on candidate protein targets of immunity) for epitope prediction (i.e., computational identification of putative epitopes) to design vaccine peptides comprising only protective epitopes, which elicit immune responses that protect against disease. This is applicable to both T- and B-cell epitopes (i.e., epitopes recognized by T cells and antibodies, respectively). Typical T-cell epitopes are proteolytically derived oligopeptides that may serve as vaccine antigens to an immune system expressing appropriate major histocompatibility complex alleles; whereas B-cell epitopes in protein antigens may adopt conformations different from those that they adopt as oligopeptides, such that recognition of a B-cell epitope by the same antibodies in both protein and oligopeptide contexts is more likely if it is intrinsically disordered (i.e., in a dynamic random coil state, rapidly sampling various conformations) and thus capable of readily undergoing conformational adjustments to enable its binding by antibody [17]. T- and B-cell epitopes may be decoupled from each other in vaccine design, as vaccines containing only T-cell epitopes can avoid antibody-mediated adverse outcomes (e.g., antibody-dependent enhancement of infection with various viral and other microbial pathogens) while vaccines containing only B-cell epitopes can elicit protective antibody responses independent of host major histocompatibility complex allele expression. For infectious disease, vaccine design based on reverse vaccinology (i.e., analysis of entire pathogen genomes to identify candidate protein targets of immunity) may be sharpened by epitope prediction to include protective epitopes while excluding nonprotective (e.g., decoy) and other potentially deleterious epitopes (e.g., mimotopes that mimic host self epitopes and can elicit autoimmune responses) [18]. Although targeting of host self epitopes is generally avoided, it may nonetheless result in net benefit among special cases of both infectious and noninfectious disease (e.g., with antibodies that block viral infection by targeting host cell surface receptors or co-receptors without causing significant host damage; or with T cells that target malignant tumor cells expressing self epitopes of nonessential host cell types such as melanocytes) [19].

Where peptide-based vaccines are designed to comprise B-cell epitopes anticipated to elicit protective immunity, antibody-type agents to the epitopes can be generated (e.g., in animals or even entirely *in vitro*) initially for passive immunization, such that any adverse reactions can be treated by administering the epitopes in peptide form to render the agents inactive, with vaccine-induced active immunization to be pursued only after safety and efficacy of passive immunization have been established [2]; and immunological adaptors can be developed to repurpose the agents for mediating immunity against other targets, with each adaptor comprising an agent-recognized B-cell epitope and a suitable target-binding moiety. Peptides also can be developed as drugs that bind to corresponding drug targets (e.g., cell surface receptors); and antibody-type agents to the drugs can be produced as corresponding antidotes and drug-concentration buffers, using essentially the same approach as employed for producing such agents to B-cell epitopes of vaccine peptides (noting that antibody-type agents also can be produced to nonpeptidic drugs by similar means) [2]. *In silico* molecular docking simulations [20] could be used to design specific target-binding immunological adaptors and drugs (both peptidic and nonpeptidic). Toward precision health, patients could be characterized by means of appropriate screening procedures (e.g., for genomic and immunological markers) to inform decisions on various possible health interventions such as vaccination, passive immunization and drug therapy on a case-by-case basis according to individual circumstances.

## Conclusion & future perspective

Planetary health entails sustainability attained through judicious resource utilization based on green chemistry. Reorientation of the pharmaceutical industry and wider health sector in line with principles of safety and sustainability can be achieved by adopting an immunocentric approach to disease control and prevention that emphasizes vaccination and other forms of immunization over xenobiotic drug therapy, to enhance immunity while avoiding environmental contamination and negative consequences thereof. This is conceivably feasible using synthetic peptides in conjunction with antibody-type agents, such that the peptides serve as vaccine components, immunological adaptors for programming of immune responses, and alternatives to nonpeptidic drugs, while the antibody-type agents serve as mediators of passive immunity, antidotes to drugs, and drug-concentration buffers. These constructs can be designed with the aid of computational tools and used to advance precision health through interventions such as immunization and drug treatment informed by individual circumstances, in part via screening for pertinent genomic and immunological markers.

Over the next 5–10 years, planetary health conceivably will emerge as the unifying analytical and decision-support framework for all human activity, from local to global levels and even beyond, further expanding and evolving into transplanetary health as space exploration is increasingly pursued. The acute sense of resource limits and attendant vulnerability on board spacecraft shall heighten and sharpen the collective human awareness of analogous constraints on Earth, thereby further driving innovation toward circular economy based on green chemistry, with peptidic agents thus being developed as ever more crucial means toward precision health for all.

**Executive summary**

- Planetary health necessitates sustainability through adaptive innovation, which is constrained by the prevailing regulatory regime based on the precautionary principle as regards safety from individual and environmental perspectives.
- Safety depends on sustainability in the long run, such that individual health needs must be satisfied while also avoiding harmful anthropogenic effects on the rest of the biosphere.
- Green chemistry provides the basis for comprehensively addressing issues of safety and sustainability through judicious resource utilization within a zero-waste circular economy.
- Antibodies, peptides and other peptidic agents can be produced and utilized for individually customized precision health in accordance with principles of green chemistry, unlike many nonpeptidic xenobiotics.
- Peptide-based vaccines and other peptidic agents for precision health can be developed on the basis of available biomolecular and other data, using immunoinformatics and other computational tools.
- Peptidic agents shall be used to an ever greater extent for immune programming and other applications toward precision health for all in the context of planetary health.

## References

[1] Hancock T (2016). Healthcare in the Anthropocene: challenges and opportunities.

[2] Caoili SE (2014). Beyond new chemical entities: advancing drug development based on functional versatility of antibodies.

[3] Kanemoto K, Moran D, Hertwich EG (2016). Mapping the carbon footprint of nations.

[4] McManus MC, Taylor CM (2015). The changing nature of life cycle assessment.

[5] Sheldon RA (2012). Fundamentals of green chemistry: efficiency in reaction design.

[6] Puckowski A, Mioduszewska K, Łukaszewicz P (2016). Bioaccumulation and analytics of pharmaceutical residues in the environment: a review.

[7] Anan H, Otomo Y, Kondo H (2016). Development of mass-casualty life support-CBRNE (MCLS-CBRNE) in Japan.

[8] Lammie SL, Hughes JM (2016). Antimicrobial resistance, food safety, and one health: the need for convergence.

[9] Tamburini S, Shen N, Wu HC, Clemente JC (2016). The microbiome in early life: implications for health outcomes.

[10] Weiss R, Scheiblhofer S, Thalhamer J (2014). Allergens are not pathogens: why immunization against allergy differs from vaccination against infectious diseases.

[11] Skwarczynski M, Toth I (2016). Peptide-based synthetic vaccines.

[12] Rader C (2014). Chemically programmed antibodies.

[13] Lawrenson SB, Arava R, North M (2017). The greening of peptide synthesis.

[14] Lloyd J, Cheyne J (2017). The origins of the vaccine cold chain and a glimpse of the future.

[15] Chandrasekhar S, Iyer LK, Panchal JP, Topp EM, Cannon JB, Ranade VV (2013). Microarrays and microneedle arrays for delivery of peptides, proteins, vaccines and other applications.

[16] Agrahari V, Agrahari V, Mitra AK (2016). Nanocarrier fabrication and macromolecule drug delivery: challenges and opportunities.

[17] Caoili SE (2015). An integrative structure-based framework for predicting biological effects mediated by antipeptide antibodies.

[18] Loomis RJ, Johnson PR (2015). Emerging vaccine technologies.

[19] Caoili SE (2014). Epitopes for protective immunity targeting antigens of pathogen and/or host (EPITAPH): toward novel vaccines against HIV and other medically challenging infections.

[20] Pagadala NS, Syed K, Tuszynski J (2017). Software for molecular docking: a review.

