# Erratum

**DOI:** 10.4155/fsoa-2017-0101e1

**Published:** 2018-04-25

In the Special Report by Salvador Eugenio Caoili, ‘Antibodies, synthetic peptides and related constructs for planetary health based on green chemistry in the Anthropocene’, which appeared in the March 2018 issue of *Future Science OA*, 4(3), FSO275 (2018), the authors name was incorrectly listed as ‘Salvador Eugenio C Caoili’. This has now been corrected to ‘Salvador Eugenio Caoili’.

The editors of *Future Science OA* would like to sincerely apologize for any inconvenience or confusion this may have caused our readers.

